# *Iris setosa* Pall. ex Link Extract Reveals Amoebicidal Activity against *Acanthamoeba castellanii* and *Acanthamoeba polyphaga* with Low Toxicity to Human Corneal Cells

**DOI:** 10.3390/microorganisms12081658

**Published:** 2024-08-13

**Authors:** Hương Giang Lê, Buyng Su Hwang, Ji-Su Choi, Yong Tae Jeong, Jung-Mi Kang, Tuấn Cường Võ, Young Taek Oh, Byoung-Kuk Na

**Affiliations:** 1 Department of Parasitology and Tropical Medicine, and Institute of Health Science, Gyeongsang National University College of Medicine, Jinju 52727, Republic of Korea; gianglee291994@gmail.com (H.G.L.); jmkang@gnu.ac.kr (J.-M.K.); vtcuong241@gmail.com (T.C.V.); 2 Department of Convergence Medical Science, Gyeongsang National University, Jinju 52727, Republic of Korea; 3 Nakdonggang National Institute of Biological Resources, Sangju 37242, Republic of Korea; hwang1531@nnibr.re.kr (B.S.H.); ccc6112@nnibr.re.kr (J.-S.C.); ytjeong@nnibr.re.kr (Y.T.J.)

**Keywords:** *Iris setosa* Pall. ex Link, *Acanthamoeba*, amoebicidal activity, programmed cell death, cysticidal activity

## Abstract

*Acanthamoeba* keratitis (AK) is a sight-threatening and difficult-to-treat ocular infection. The significant side effects of current AK treatments highlight the urgent need to develop a safe and effective AK medication. In this study, the amoebicidal activity of *Iris setosa* Pall. ex Link extract (ISE) against *Acanthamoeba* was examined and its specific amoebicidal mechanism was explored. ISE induced significant morphological changes in *Acanthamoeba* trophozoites and exhibited amoebicidal activity against *A. castellanii* and *A. polyphaga*. ISE was further fractionated into five subfractions by sequential extraction with *n*-hexane, chloroform, ethyl acetate, *n*-butanol, and water, and their amoebicidal activities and underlying amoebicidal mechanisms were investigated. The *n*-butanol subfraction of ISE (ISE-BuOH) displayed selective amoebicidal activity against the *Acanthamoeba* species with minimal cytotoxicity in human corneal cells (HCE-2). ISE-BuOH triggered apoptosis-like programmed cell death (PCD) in amoebae, characterized by DNA fragmentation, increased ROS production, and caspase-3 activity elevation. ISE-BuOH also demonstrated a partial cysticidal effect against the amoeba species. ISE-BuOH could be a promising candidate in the development of therapeutic drugs for AK.

## 1. Introduction

*Acanthamoeba* keratitis (AK) is an ocular disease resulting from opportunistic infections by *Acanthamoeba* species [1]. When amoebae invade the corneal epithelium, they infiltrate the corneal stroma, leading to inflammation and substantial damage to the corneal lamellae, which can result in severe visual impairment or vision loss and even blindness [1,2]. Contact lens wearers who do not adhere to sanitary practices are at a high-risk for AK, and the incidence of global AK cases has been on the rise. This increase is correlated with the growing number of contact lens users [1]. AK is a progressive, chronic condition; hence, a prompt and accurate diagnosis in the initial stages of infection, along with the appropriate treatment, is imperative to avoid extensive corneal damage and ensure the complete recovery of vision. However, diagnosing AK can be difficult because of the nonspecific clinical symptoms initially presented [1,2].

The absence of effective therapeutics poses a significant concern for treating AK. Low concentrations of antiseptic agents like polyhexamethylene biguanide and chlorhexidine (CHX) represent the primary treatment for AK. Both agents are effective against trophozoites and cysts of *Acanthamoeba* by inducing structural damages in the amoebae, but they can harm corneal cells and keratocytes at clinically relevant doses [3,4,5]. A relatively high failure rate of antiseptics was also reported, probably due to their inadequate penetration into the cornea [6]. While topical antifungals, antibacterials, antivirals, and even corticosteroids are also prescribed, their use often results in an initial improvement followed by a deterioration of clinical signs [7]. Given the limited availability of effective AK medications, there is a critical need to develop safe and effective therapeutics for AK.

Numerous studies have been conducted to identify AK therapeutics among natural products, and a variety of natural compounds and extracts with activity against *Acanthamoeba* have been identified from various plants [8,9,10,11,12,13,14,15,16,17,18]. Promising amoebicidal and antiamoebic properties of natural compounds or plant extracts against the pathogenic *Acanthamoeba* species have expanded the potential to develop AK drugs using natural substances, yet additional studies on their safety and clinical efficacy are needed.

To discover natural compounds with anti-*Acanthamoeba* activity in plants, we conducted a mass screening of over 300 plant extracts from wild plants in Korea and identified that *Iris setosa* Pall. ex Link extract (ISE) demonstrated significant amoebicidal activity against the *Acanthamoeba* species. *Iris setosa* Pall. ex Link is a rhizomatous geophyte that belongs to the Genus *Iris* and is widely distributed in northern temperate zones, such as North America and East Asia. The Genus *Iris* is utilized in the food industry and for medicinal applications across many countries, owing to its notable pharmacological properties. Phytochemical studies have shown that the *Iris* species contains various bioactive compounds, indicating their potential for pharmacological applications [19,20,21]. These compounds have been reported to confer several beneficial effects on human health, including anticancer, antioxidant, anti-inflammatory, and antiplasmodial properties [22,23,24,25,26]. In this study, the amoebicidal activity of ISE against the *Acanthamoeba* species was thoroughly analyzed, and the molecular mechanisms underlying its amoebicidal properties were investigated.

## 2. Materials and Methods

### 2.1. Preparation of ISE and ISE Subfractions

The whole body of *Iris setosa* Pall. ex Link was gathered in Buyeo, Korea (126.9634° E, 36.3169° N) in May 2019. The plants were identified by a taxonomy expert using the *Iris* classification keys. A voucher specimen (NNIBRVP59234) was deposited at the Library of the Nakdonggang National Institute of Biological Resources (Sangju, Korea). The plants were thoroughly washed with distilled water multiple times to eliminate adhesive impurities and were then air-dried. Dried plants were subsequently ground using an electrical blender. The ethanolic extract from the plants was obtained by macerating 2.24 kg of ground plants in 70% aqueous ethanol at room temperature (RT) for 2 days, followed by sonication and filtration using filter paper (CHMLAB, Terrassa, Barcelona, Spain) (Supplementary Materials: Figure S1). The organic solvent from the sample was evaporated using a rotary vacuum evaporator. The resulting crude extract (named ISE, 173.5 g, yield 7.7%) was then sequentially subfractionated into *n*-hexane (HX), chloroform (CHCl_3_), ethyl acetate (EA), *n*-butanol (BuOH), and distilled water (H_2_O) layers. Each layer was centrifuged, filtered, and lyophilized respectively (Supplementary Materials: Figure S1). The obtained dried fractions (named ISE-HX, ISE-CHCl_3_, ISE-EA, ISE-BuOH, and ISE-H_2_O) were preserved at −20 °C until analysis.

### 2.2. Cultivation of Acanthamoeba and Human Corneal Epithelium Cells

*Acanthamoeba castellanii* (ATCC-30868; American Type Culture Collection, Manassas, VA, USA) and *Acanthamoeba polyphaga* (ATCC-30461; American Type Culture Collection) were cultured in a peptone–yeast–glucose (PYG) medium at a temperature of 25 °C. Human corneal epithelium cells (HCE-2; ATCC CRL-11135, American Type Culture Collection) were maintained at 37 °C in a humidified atmosphere containing 5% CO_2_, using the methodology previously described [17,18].

### 2.3. Amoebicidal Assay

Dried ISE and its subfractions were dissolved in 100% dimethyl sulfoxide (DMSO; Sigma, St. Louis, MO, USA) to prepare stock solutions at 20 mg/mL. The amoebicidal activity of ISE and its subfractions was evaluated using a 96-well microplate, following previously described methods [17,18]. Various concentrations (0–200 μg/mL) of ISE or its subfractions were added to amoebae (5 × 10^4^ cells) cultured in a 96-well microplate and incubated at 25 °C for 48 h. Amoebae treated solely with 0.1% DMSO (Sigma, St. Louis, MO, USA), which matched the dilution concentration used for ISE and its subfractions, served as controls. Morphological changes in amoebae were observed microscopically. The viability of the amoebae was assessed using a CellTiter-Blue^®^ Cell Viability Assay (Promega, Madison, WI, USA). Viability values were ascertained using GraphPad Prism version 9.1.0 (GraphPad Software, San Diego, CA, USA). The 50% and 90% inhibitory concentrations (IC_50_ and IC_90_, respectively) of ISE and ISE-BuOH against amoebae were computed using GraphPad Prism version 9.1.0 (GraphPad Software, San Diego, CA, USA).

### 2.4. Cytotoxicity Assay for HCE-2 Cells

The cytotoxic potential of ISE and its subfractions on HCE-2 cells was evaluated through a cell viability assay, as previously detailed [17,18]. Serial dilutions of ISE or its subfractions, ranging from 0 to 200 μg/mL, were applied to the cells seeded in a 96-well microplate and then incubated for 48 h. Changes in cell morphology were observed using microscopic examination. Cell viability was determined using a CellTiter-Blue^®^ Cell Viability Assay (Promega, Madison, WI, USA). As controls, HCE-2 cells were exposed to 0.1% DMSO, which has been confirmed not to induce any morphological changes or cell death [17]. Using GraphPad Prism version 9.1.0 (GraphPad Software, San Diego, CA, USA), the 50% cytotoxic concentration (CC_50_; the concentration required to reduce cell viability by 50%) and the sensitivity index (SI; calculated as CC_50_/IC_50_) of ISE-BuOH were calculated.

### 2.5. Apoptosis/Necrosis Assay

To determine if ISE-BuOH initiates apoptosis/necrosis in *A. castellanii* and *A. polyphaga* trophozoites, we utilized an Apoptosis/Necrosis Detection Kit (Abcam, Cambridge, UK) to analyze such events in amoebae exposed to ISE-BuOH. Upon treating the amoebae (5 × 10^4^ cells) with ISE-BuOH (IC_90_), they were incubated in a 96-well black/clear bottom plate (Thermo Fisher Scientific, Waltham, MA, USA) at 25 °C for 24 or 48 h. As a control, amoebae were treated with only 0.1% DMSO (Sigma, St. Louis, MO, USA) and 0.02% CHX (Sigma, St. Louis, MO, USA), serving as negative and positive controls, respectively. Subsequently, CytoCalcein, Apopxin Green, and 7-aminoactinomycin D (7-AAD) were added to the amoebae and incubated at RT for 1 h in the dark. The amoebae were then washed twice with phosphate buffered saline (PBS, pH 7.4), followed by the assessment of fluorescence signals using an EVOS M5000 Imaging System (Thermo Fisher Scientific, Waltham, MA, USA): the 4′,6-diamidino-2-phenylindole (DAPI) channel (405 and 450 nm) for CytoCalcein, the green fluorescent protein (GFP) channel (490 and 525 nm) for Apopxin Green, and the Texas Red channel (550 and 650 nm) for 7-AAD.

### 2.6. Terminal Deoxynucleotidyl Transferase-Mediated dUTP Nick End-Labeling (TUNEL) Assay

To further validate the apoptotic event in *Acanthamoeba* upon treatment with ISE-BuOH, DNA fragmentation was analyzed by a TUNEL assay. *A. castellanii* and *A. polyphaga* trophozoites (10^6^ cells) were seeded in each well of a 6-well microplate (Thermo Fisher Scientific, Waltham, MA, USA) and incubated at 25 °C overnight. After adding ISE-BuOH (IC_90_) to the amoebae, the plate was incubated at 25 °C for 24 or 48 h. The cells were subsequently harvested, fixed in 3.7% formaldehyde at RT for 20 min, and immersed in 70% cold ethanol for 30 min. Amoebae were double-stained with TUNEL and propidium iodide (PI) using an In Situ Direct DNA Fragmentation (TUNEL) Assay Kit (Abcam, Cambridge, UK). Amoebae not treated with ISE-BuOH served as a negative control. Fluorescence within the cells was examined using a EVOS M5000 Imaging System (Thermo Fisher Scientific, Waltham, MA, USA): the GFP channel for TUNEL, and the Texas Red channel for PI.

### 2.7. Intracellular Reactive Oxygen Species (ROS) Assay

Change in intracellular ROS generation in ISE-BuOH-treated *Acanthamoeba* was assessed using a 2′,7′-Dichlorofluorescein diacetate (DCFDA)/H2DCFDA Cellular ROS Assay Kit (Abcam, Cambridge, UK). Amoebae (5 × 10^4^ cells) seeded in a black/clear bottom 96-well microplate (Thermo Fisher Scientific, Waltham, MA, USA) received treatment with ISE-BuOH (IC_90_) and were incubated for either 24 or 48 h. Amoebae treated with 0.1% DMSO (Sigma) and 0.02% (Sigma) served as negative and positive controls, respectively. Following incubation, amoeba cells were rinsed with PBS and stained using a 20 µM DCFDA solution at 25 °C for 45 min in darkness. Afterward, the cells were washed three times with PBS, and the fluorescence signals were captured using a EVOS M5000 Imaging System (Thermo Fisher Scientific, Waltham, MA, USA) in the GFP channel. Additionally, fluorescence was measured at excitation and emission wavelengths of 480 nm and 530 nm, respectively, using a VICTOR Nivo Multimode Microplate Reader (PerkinElmer, Waltham, MA, USA).

### 2.8. Mitochondrial Membrane Potential (MMP) Assay

Electrochemical gradient changes across mitochondrial membranes in amoebae were assessed utilizing a JC-1 Assay Kit (Abcam, Cambridge, UK). *A. castellanii* and *A. polyphaga* trophozoites (5 × 10^4^ cells) were plated in each well of a 96-well black/clear bottom plate (Thermo Fisher Scientific, Waltham, MA, USA) and incubated at 25 °C overnight. ISE-BuOH (IC_90_) was subsequently added, and the plate was incubated at 25 °C for either 24 or 48 h. Cells were then washed with PBS, and JC-1 (1 μM) was added to each well. The plate was incubated at RT for 30 min in the dark. Green and red fluorescence was captured using an EVOS M5000 Imaging System (Thermo Fisher Scientific, Waltham, MA, USA) in the GFP channel for the JC-1 monomer and the Texas Red channel for the JC-1 aggregated form. Fluorescence release was also measured at Ex/Em = 480/530 nm for the JC-1 monomer and Ex/Em = 530/620 for the JC-1 aggregated form using a VICTOR Nivo Multimode Microplate Reader (PerkinElmer, Waltham, MA, USA). Amoebae treated with 0.1% DMSO (Sigma) and 0.02% CHX (Sigma) served as negative and positive controls, respectively.

### 2.9. ATP Assay

ATP production levels in ISE-BuOH- treated and -untreated *A. castellanii* were assessed using a Luminescent ATP Detection Kit (Abcam, Cambridge, UK). *A. castellanii* and *A. polyphaga* trophozoites (5 × 10^4^ cells) were each seeded in a 96-well white/clear bottom plate (Thermo Fisher Scientific, Waltham, MA, USA) and incubated at 25 °C overnight. ISE-BuOH (IC_90_) was administered to the amoebae and further incubated at 25 °C for 24 or 48 h. Cells were lysed using detergent to stabilize the ATP, followed by the addition of the substrate solution. The mixture was incubated for 10 min in darkness, followed by luminescence measurement using a VICTOR Nivo Multimode Microplate Reader (PerkinElmer, Waltham, MA, USA). Control amoeba cells were treated with 0.1% DMSO (Sigma) and 0.02% CHX (Sigma).

### 2.10. Caspase-3 Assay

*A. castellanii* and *A. polyphaga* trophozoites (5 × 10^4^ cells) were each seeded in wells of a black/clear bottom 96-well plate (Thermo Fisher Scientific, Waltham, MA, USA) and incubated at 25 °C overnight. The controls included amoebae treated with 0.1% DMSO (Sigma). ISE-BuOH (IC_90_) was added to amoebae and incubated for 24 or 48 h. A NucView^®^ 488 caspase-3 substrate (Biotium, Fremont, CA, USA) was subsequently added as per the manufacturer’s protocol, and amoebae were further incubated at 25 °C for 1 h in darkness. After thrice washing with buffer, fluorescence signals were detected using an EVOS M5000 Imaging System (Thermo Fisher Scientific, Waltham, MA, USA) on the GFP channel.

### 2.11. Cysticidal Activity Assay

*Acanthamoeba* trophozoites cultured in PYG medium were encysted using a previous method [27]. Cyst formation was verified via microscopic examination, and mature cysts were isolated by treating them with 0.5% sodium dodecyl sulfate (Sigma, St. Louis, MO, USA) for 10 min. These mature cysts were collected by centrifugation and washed three times with PBS. Mature cysts (5 × 10^5^) were then placed in a 24-well microplate (Thermo Fisher Scientific, Waltham, MA, USA) with ISE-BuOH (IC_90_) for either 24 or 48 h. For positive controls, amoeba cysts treated with 0.02% CHX (Sigma) were employed. Mature cysts not treated with ISE-BuOH or 0.02% CHX (Sigma) served as controls. After three PBS washes, mature cysts were excysted in a PYG medium supplemented with 20% fetal bovine serum (Gibco, Grand Island, NY, USA). By the fourth day post-excystation, amoebae were harvested by centrifugation, and the numbers of non-excysted dead cysts and live trophozoites were quantified using the trypan blue staining method [18,28].

### 2.12. Statistical Analysis

All assays were conducted in triplicate, yielding results as mean ± standard deviation (SD) for three separate assays. Statistical evaluations were performed using GraphPad Prism version 9.1.0 (GraphPad Software), with statistical significance determined by a one-way analysis of variance and Dunnett’s post hoc test. A *p* value < 0.05 was deemed statistically significant.

## 3. Results

### 3.1. ISE Showed Amoebicidal Activity against Acanthamoeba Trophozoites

**Figure 1:**
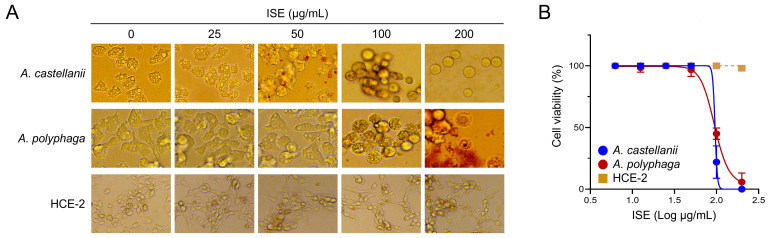
Amoebicidal activity of ISE against *Acanthamoeba* trophozoites. (**A**) Morphological changes. Both *Acanthamoeba* species exhibited morphological alterations after treatment with ISE in a dose-dependent manner. (**B**) Cell viability. The viability of both amoebae and HCE-2 cells is presented as a percentage relative to the negative controls that were not treated with ISE. Results represent the mean ± SD (error bars) from three independent experiments.

ISE demonstrated amoebicidal efficacy against both *A. castellanii* and *A. polyphaga* trophozoites in a concentration-dependent manner (Figure 1). Compared to the untreated controls, amoebae treated with ISE displayed significant morphological changes. Moreover, the viability of amoebae decreased in a dose-dependent manner following ISE exposure. Concurrently, HCE-2 cell viability was minimally impacted by ISE, even though mild morphological alterations were noted at higher ISE concentrations (Figure 1). The IC_50_ and IC_90_ indices of ISE for *A. castellanii* were 95.43 ± 1.27 µg/mL and 101.58 ± 2.10 µg/mL, respectively. Meanwhile, the IC_50_ and IC_90_ values of ISE for *A. polyphaga* were slightly higher, registering at 97.09 ± 0.57 and 146.76 ± 1.02 µg/mL, respectively. These results indicate that ISE exhibits amoebicidal activity against *Acanthamoeba* trophozoites while demonstrating low or negligible cytotoxicity to HCE-2 cells.

### 3.2. ISE-BuOH Showed Selective Amoebicidal Activity against Acanthamoeba Trophozoites

**Figure 2:**
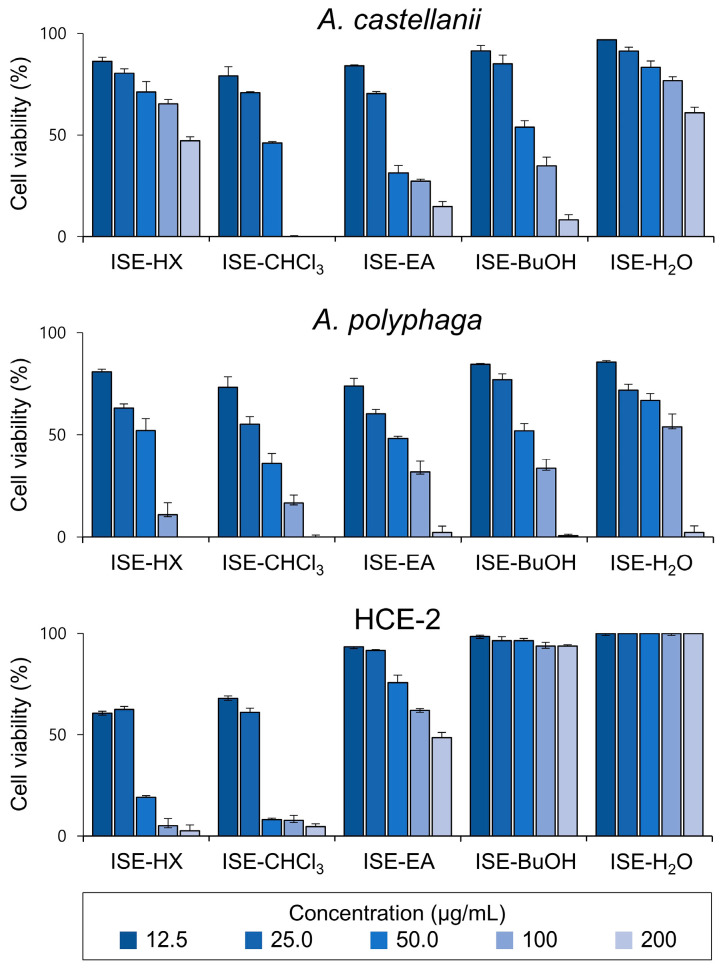
Amoebicidal activity of ISE subfractions against *Acanthamoeba* trophozoites. Various concentrations of ISE subfractions were administered to *A. castellanii* and *A. polyphaga* trophozoites, and the amoebicidal activity was evaluated. HCE-2 cells were included in the assay to assess the potential cytotoxicity of the ISE subfractions. The viability of both the amoebae and human cells is expressed as a percentage relative to negative controls that were not treated with ISE subfraction. The results are presented as mean ± SD (error bars) from three independent experiments.

**Figure 3:**
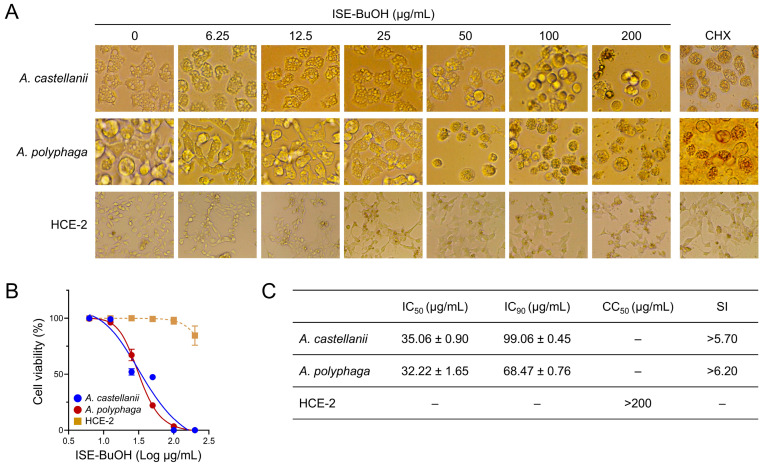
Amoebicidal activity of ISE-BuOH against *Acanthamoeba* trophozoites. (**A**) Morphological changes. (**B**) Cell viability assay. The viability of both the amoebae and HCE-2 cells is presented as a percentage relative to negative controls that were not treated with ISE-BuOH. Results are shown as mean ± SD (error bars) from three independent experiments. (**C**) Summary. IC_50_, IC_90_, CC_50_, and SI values were derived from data collected over three independent assays.

To further explore the amoebicidal effect of ISE, it was fractionated into five subfractions, and their amoebicidal activities were assessed (Figure 2). Of these, four subfractions, ISE-HX, ISE-CHCl_3_, ISE-EA, and ISE-BuOH, exhibited amoebicidal activity. In contrast, ISE-H_2_O did not display notable amoebicidal activity when compared with other subfractions. While ISE-HX and ISE-CHCl_3_ showed potent amoebicidal activities, they also caused strong cytotoxicity in HCE-2 cells. ISE-EA proved effective against amoebae but also exhibited considerable cytotoxic effects on HCE-2 cells at higher concentrations. Conversely, ISE-BuOH manifested a selective amoebicidal activity against both *Acanthamoeba* trophozoites in a dose-dependent manner, with minimal or no significant impact on the viability of HCE-2 cells (Figure 2 and Figure 3). The IC_50_ values for ISE-BuOH targeting *A. castellanii* and *A. polyphaga* were identified to be 35.06 ± 0.90 and 32.22 ± 1.65 μg/mL, respectively. The CC_50_ value was >200 µg/mL (Figure 3). The SI values for ISE-BuOH against *A. castellanii* and *A. polyphaga* were >5.7 and >6.2, respectively, underscoring its selective amoebicidal effectiveness.

### 3.3. ISE-BuOH Induced Apoptosis-like Programmed Cell Death (PCD) in A. castellanii and A. polyphaga Trophozoites

**Figure 4:**
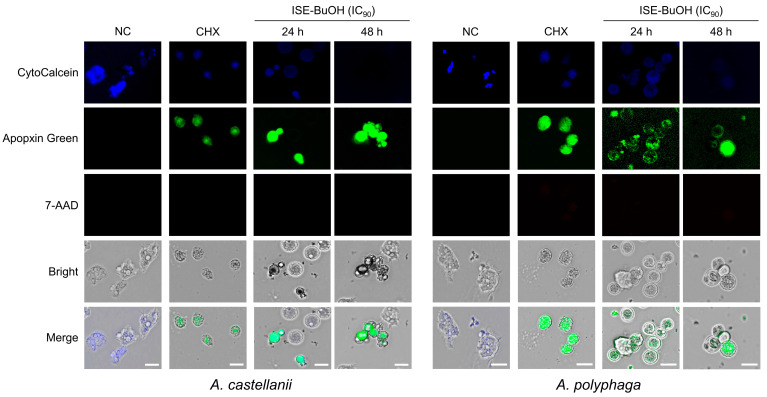
Apoptosis/necrosis assay. The fluorescence staining assay was conducted on *A. castellanii* and *A. polyphaga* trophozoites after treatment with ISE-BuOH. NC, negative controls treated with the same concentration of DMSO without ISE-BuOH for 24 h. CHX, positive controls treated with 0.02% CHX for 24 h. Scale bars: 10 μm.

**Figure 5:**
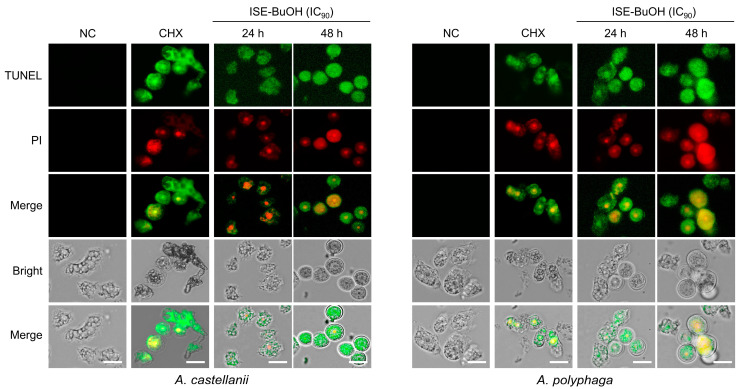
TUNEL assay. DNA fragmentation was detected in *A. castellanii* and *A. polyphaga* trophozoites treated with ISE-BuOH. Cells were counterstained with TUNEL (green) and propidium iodide (PI, red). NC, negative control amoebae treated with the same concentration of DMSO without ISE-BuOH for 24 h. CHX, positive controls treated with 0.02% CHX for 24 h. Scale bars: 10 μm.

*A. castellanii* and *A. polyphaga* trophozoites exhibited apoptosis events following treatment with ISE-BuOH (Figure 4). ISE-BuOH-untreated amoebae exhibited a blue CytoCalcein fluorescence, indicating the absence of apoptotic events in the amoebae. In contrast, in amoebae treated with ISE-BuOH, the blue fluorescence was absent, whereas the green fluorescence of Apopxin Green increased in a time- and dose-dependent manner, suggesting the occurrence of apoptotic events. No red 7-AAD fluorescence, which indicates necrosis, was detected in the amoebae treated with ISE-BuOH. To further validate the apoptosis events in ISE-BuOH-treated *A. castellanii* and *A. polyphaga*, DNA fragmentation in the treated amoebae was analyzed using a TUNEL assay. Both the green fluorescence of TUNEL and the red fluorescence of PI, indicating DNA fragmentation, showed increased levels in the ISE-BuOH-treated amoebae in time- and dose-dependent manners (Figure 5). These findings indicated that ISE-BuOH induced apoptosis-like PCD in both *Acanthamoeba* species.

### 3.4. ISE-BuOH Enhanced ROS Production in A. castellanii and A. polyphaga Trophozoites

**Figure 6:**
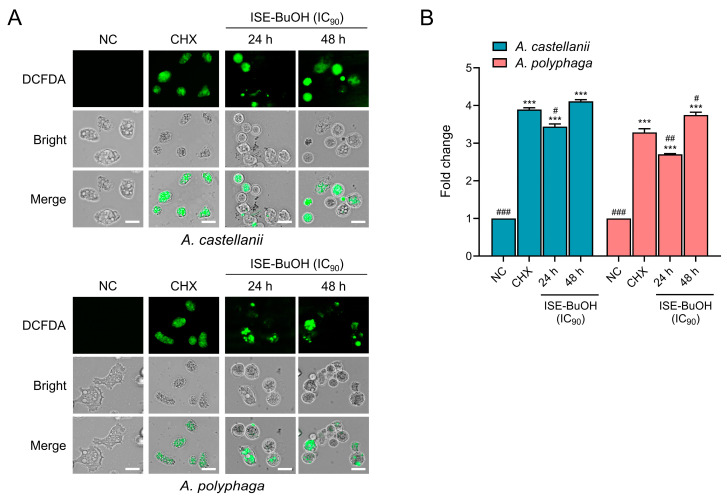
ISE-BuOH enhanced intracellular ROS generation in *A. castellanii* and *A. polyphaga*. (**A**) Microscopic examination. The images shown are representative of the cell population observed across three individual experiments. NC represents the negative controls that did not receive ISE-BuOH treatment. CHX serves as the positive controls, treated with 0.02% CHX for 24 h. Scale bars: 10 μm. (**B**) Fluorometric analysis. Results are presented as mean ± SD (error bars) from three independent assays. Significance levels are indicated as follows: *** *p* < 0.0001 compared to NC; ### *p* < 0.0001, ## *p* < 0.001, and # *p* < 0.01 compared to CHX.

ISE-BuOH treatment resulted in increased intracellular ROS generation in *A. castellanii* and *A. polyphaga* trophozoites, as evidenced by enhanced green fluorescence related to ROS, compared to the negative controls, in a time- and dose-dependent manner (Figure 6).

### 3.5. ISE-BuOH Compromised Mitochondrial Function in A. castellanii and A. polyphaga Trophozoites

**Figure 7:**
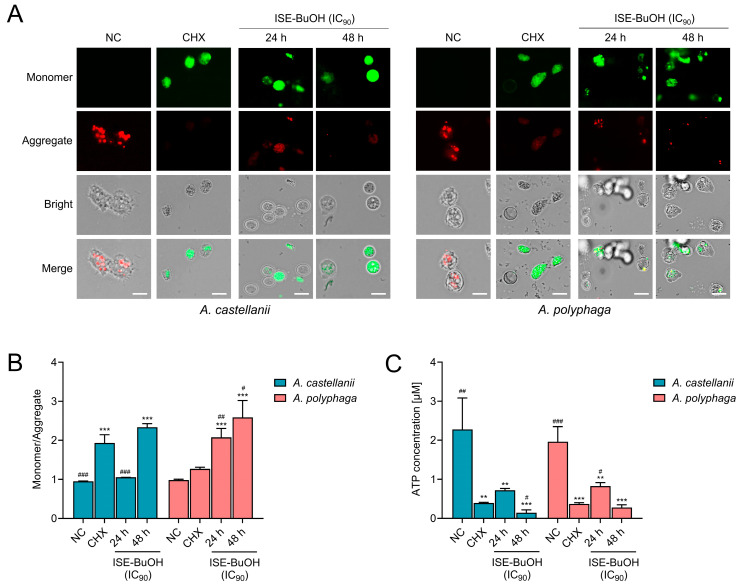
ISE-BuOH induced mitochondrial dysfunction in *A. castellanii* and *A. polyphaga*. (**A**) Microscopic examination. Amoeba cells exposed to ISE-BuOH exhibited green fluorescence (monomers), indicative of MMP collapse. CHX, positive controls treated with 0.02% CHX for 24 h. Scale bars: 10 μm. (**B**) Fluorometric assay. Results represent the mean ± SD (error bars) from three independent trials. (**C**) ATP assay. A notable decrease in ATP production in amoebae treated with ISE-BuOH (IC_90_) was detected. The findings represent the mean ± SD (error pages) from three independent experiments. *** *p* < 0.0001 and ** *p* < 0.001 compared to NC. ### *p* < 0.0001, ## *p* < 0.001, and # *p* < 0.01 compared to CHX.

Untreated *A. castellanii* and *A. polyphaga* exhibited red JC-1 aggregate fluorescence, reflecting the maintenance of normal MMP (Figure 7A,B). In contrast, treatment with ISE-BuOH led to an increase in green fluorescence, indicating the formation of JC-1 monomers in the amoebae, suggesting MMP depolymerization in a time- and dose-dependent manner (Figure 7A,B). Additionally, a marked reduction in ATP production in ISE-BuOH-treated amoebae was noted (Figure 7C). These results implied that ISE-BuOH caused mitochondrial damage and dysfunction in amoebae.

### 3.6. ISE-BuOH Increased Caspase-3 in A. castellanii and A. polyphaga Trophozoites

**Figure 8:**
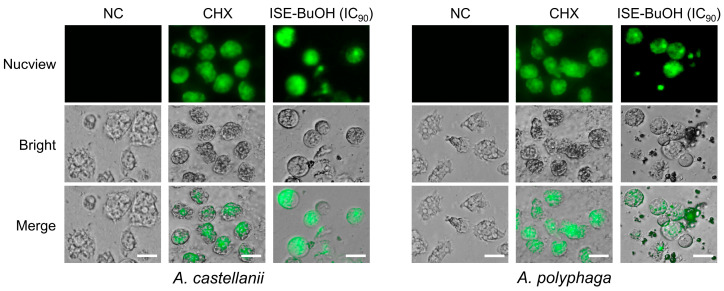
ISE-BuOH enhanced caspase-3 activity in *A. castellanii* and *A. polyphaga*. Green fluorescence, indicating increased caspase-3 activity, was observed in ISE-BuOH (IC_90_)-treated amoebae. For the positive controls, 0.02% CHX was used. NC refers to the negative controls treated with 0.1% DMSO without ISE-BuOH. Scale bars: 10 μm.

To further analyze the association of apoptosis in the amoebicidal activity of ISE-BuOH, the increase in caspase-3 in the amoebae was examined. Green fluorescence, indicative of caspase-3 activation, was observed in *A. castellanii* and *A. polyphaga* trophozoites following ISE-BuOH treatment. A comparable green signal was detected in amoebae treated with 0.02% CHX, whereas amoebae not treated with ISE-BuOH exhibited no such signal (Figure 8).

### 3.7. ISE-BuOH Revealed Partial Cysticidal Activity

**Figure 9:**
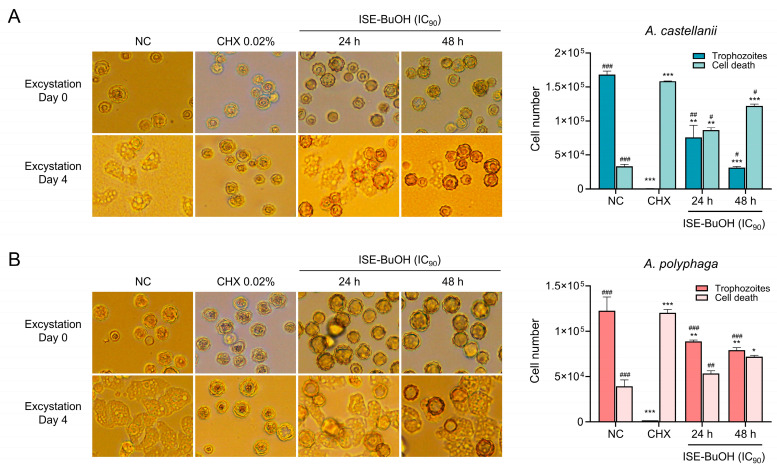
Cysticidal activity of ISE-BuOH. Encystation of mature cysts of *A. castellanii* and *A. polyphaga* were inhibited by the pretreatment of ISE-BuOH (IC_90_). Mature cysts treated with 0.02% CHX for 24 h were used as positive controls. NC denotes negative controls, which did not receive ISE-BuOH or 0.02% CHX treatment. (**A**) Microscopic examination. (**B**) Comparative analysis of the numbers of dead cysts and live trophozoites in amoebae. *** *p* < 0.0001, ** *p* < 0.001, and * *p* < 0.01 compared to NC. ### *p* < 0.0001, ## *p* < 0.001, and # *p* < 0.01 compared to CHX.

Most mature cysts of *A. castellanii* and *A. polyphaga* excysted to trophozoites by day 4 post-excystation (Figure 9). In contrast, mature cysts treated with 0.02% CHX did not excyst, indicating cyst death. Treatment with ISE-BuOH partially inhibited the excystation of mature cysts to viable trophozoites in *A. castellanii* and *A. polyphaga*. This intervention led to an increase in cysts that failed to excyst over time, resulting in a reduction in trophozoite numbers (Figure 9).

## 4. Discussion

ISE and ISE-BuOH demonstrated significant amoebicidal effects against *Acanthamoeba* trophozoites, while exhibiting minimal toxicity to human corneal cells. High SI values for ISE-BuOH against both *A. castellanii* and *A. polyphaga* trophozoites underscored its selective amoebicidal activity, enhancing its potential as a safe AK drug candidate. We further investigated the underlying amoebicidal mechanism of ISE-BuOH. Notable cellular events associated with apoptosis-like PCD, such as DNA fragmentation, mitochondrial dysfunction, and the activation of caspase family enzymes [29,30,31,32,33,34], were observed following ISE-BuOH treatment, suggesting that ISE-BuOH promotes amoebicidal activity in *Acanthamoeba* by inducing apoptosis-like PCD. Apoptosis-like PCD in pathogenic free-living amoebae, such as *Acanthamoeba* and *Naegleria fowleri*, triggered by plant extracts or natural compounds, has been previously reported [13,16,17,18,35]. Similar to the previous studies, ISE-BuOH also caused an increase in intracellular ROS generation in the amoebae, which may have subsequently facilitated mitochondrial dysfunction and DNA fragmentation, resulting in the apoptosis-like PCD of *Acanthamoeba*. A comprehensive examination of the in-depth molecular mechanisms or pathways of apoptosis-like PCD in *Acanthamoeba* trophozoites triggered by ISE-BuOH is warranted, but these findings highlight ISE-BuOH as a potential therapeutic or adjunctive agent for AK.

The notable pharmacological benefits of the Genus *Iris* have been largely investigated. A wide array of secondary metabolites or bioactive compounds derived from the *Iris* species exhibits diverse biological activities, such as antibacterial, antiprotozoal, antioxidant, anticancer, and anti-inflammatory properties [36,37,38,39,40,41,42,43]. However, the composition of these bioactive compounds in plants is subject to variation due to a variety of ecological factors, and standardizing raw plant materials remains a challenge. A limitation of this study is that a single compound with amoebicidal activity from ISE-BuOH could not be effectively isolated and characterized despite repeated trials. This subfraction likely comprises few or multiple compounds. However, ISE-BuOH revealed substantial amoebicidal activity of IC_50_ values of 35.06 ± 0.90 and 32.22 ± 1.65 μg/mL for the trophozoites of *A. castellanii* and *A. polyphaga*, respectively. These values were comparable to or lower than those of other plant extracts previously reported [44,45,46,47]. Therefore, it is essential to continue research efforts to isolate and define the specific compound responsible for amoebicidal activity in ISE-BuOH.

One major challenge in treating AK is the biological characteristics of *Acanthamoeba* that enable it to form cysts with a double-layered wall. The *Acanthamoeba* cyst wall, composed of β-glucan and other complex polysaccharides [48,49] provides significant resistance against chemical and physical stress. Many disinfecting solutions used for contact lenses prove insufficient against amoeba cysts [50]. *Acanthamoeba* trophozoites can transform into cysts and persist within corneal tissue, leading to recurrent infections through excystation. Thus, it is crucial for AK treatments to effectively eradicate both amoeba trophozoites and cysts. While ISE-BuOH shows promising amoebicidal activity against trophozoites, it only partially impacted *Acanthamoeba* cysts, probably due to limited permeability across the thick cyst walls. A similar phenomenon was also identified in plant extracts or natural compounds, which showed antiamoebic activity against *Acanthamoeba* [18,44]. Investigating synergistic effects with other drugs that exhibit cysticidal activity could further enhance the effectiveness of ISE-BuOH.

## 5. Conclusions

This study suggested that ISE-BuOH exerts promising amoebicidal activity against *Acanthamoeba* trophozoites by inducing apoptosis-like PCD in amoebae. Partial cysticidal activity in ISE-BuOH was also observed. These findings provide a basis for considering ISE-BuOH as a potential alternative therapeutic or complementary compound for AK treatment, with reduced toxicity against corneal cells. Further investigations into the detailed amoebicidal molecular mechanisms of ISE-BuOH, and the identification of its single active amoebicidal components, are required. An in vivo study to assess the therapeutic efficacy of ISE-BuOH should also be conducted.

## Data Availability

The data supporting the conclusions of this article are provided within the article. The original data in the present study are available from the corresponding authors upon request.

## Supplementary Materials

The following supporting information can be downloaded at: https://www.mdpi.com/article/10.3390/microorganisms12081658/s1, Figure S1: Preparations of ISE and its subfractions.; Figure S2: Effect of ISE-BuOH on HCE-2 cells.
