# Supplementary material for: Iris setosa Pall. ex Link Extract Reveals Amoebicidal Activity against Acanthamoeba castellanii and Acanthamoeba polyphaga with Low Toxicity to Human Corneal Cells
Source: Microorganisms. 2024 Aug 13;12(8):1658. doi: 10.3390/microorganisms12081658 (PMC11356916; doi:10.3390/microorganisms12081658)

Supplement Files

Supplement File 1: Figure S1. Preparations of ISE and its subfractions.

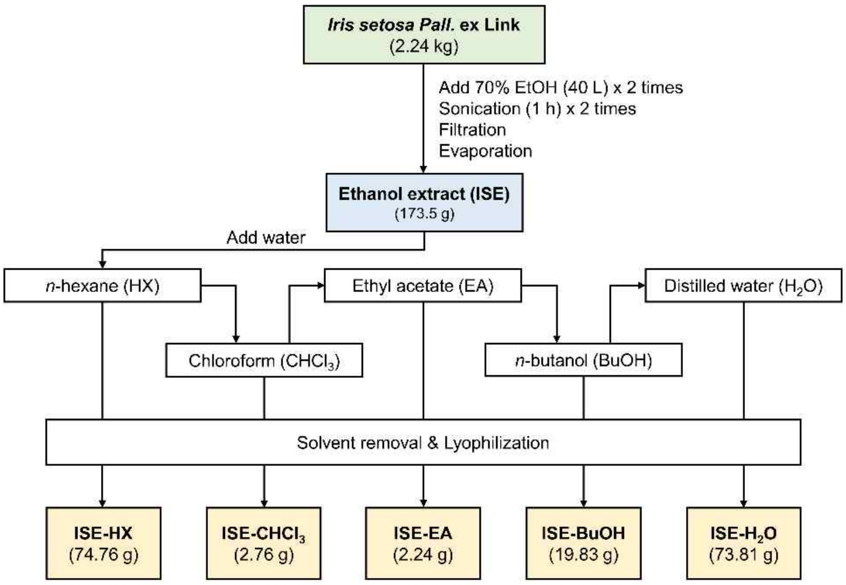

Supplement File 2: Figure S2. Effect of ISE-BuOH for HCE-2 cells. (**A**) Apoptosis/necrosis assay. (**B**) TUNEL assay. (**C**) Intracellular ROS assay. NC, negative control without treatment of ISE-BuOH.

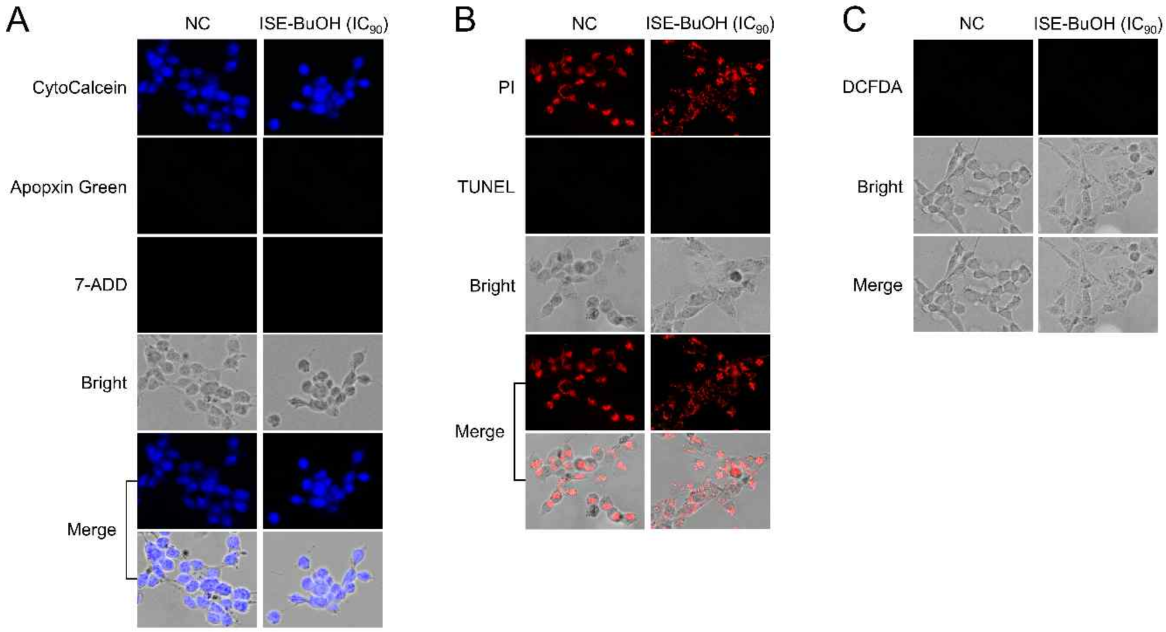

Supplement: Supplementary file 1 [file microorganisms-12-01658-s001.zip › Supplemental files.pdf]
