# Supplementary figures and images for: Iris setosa Pall. ex Link Extract Reveals Amoebicidal Activity against Acanthamoeba castellanii and Acanthamoeba polyphaga with Low Toxicity to Human Corneal Cells
Source: Microorganisms. 2024 Aug 13;12(8):1658. doi: 10.3390/microorganisms12081658 (PMC11356916; doi:10.3390/microorganisms12081658)

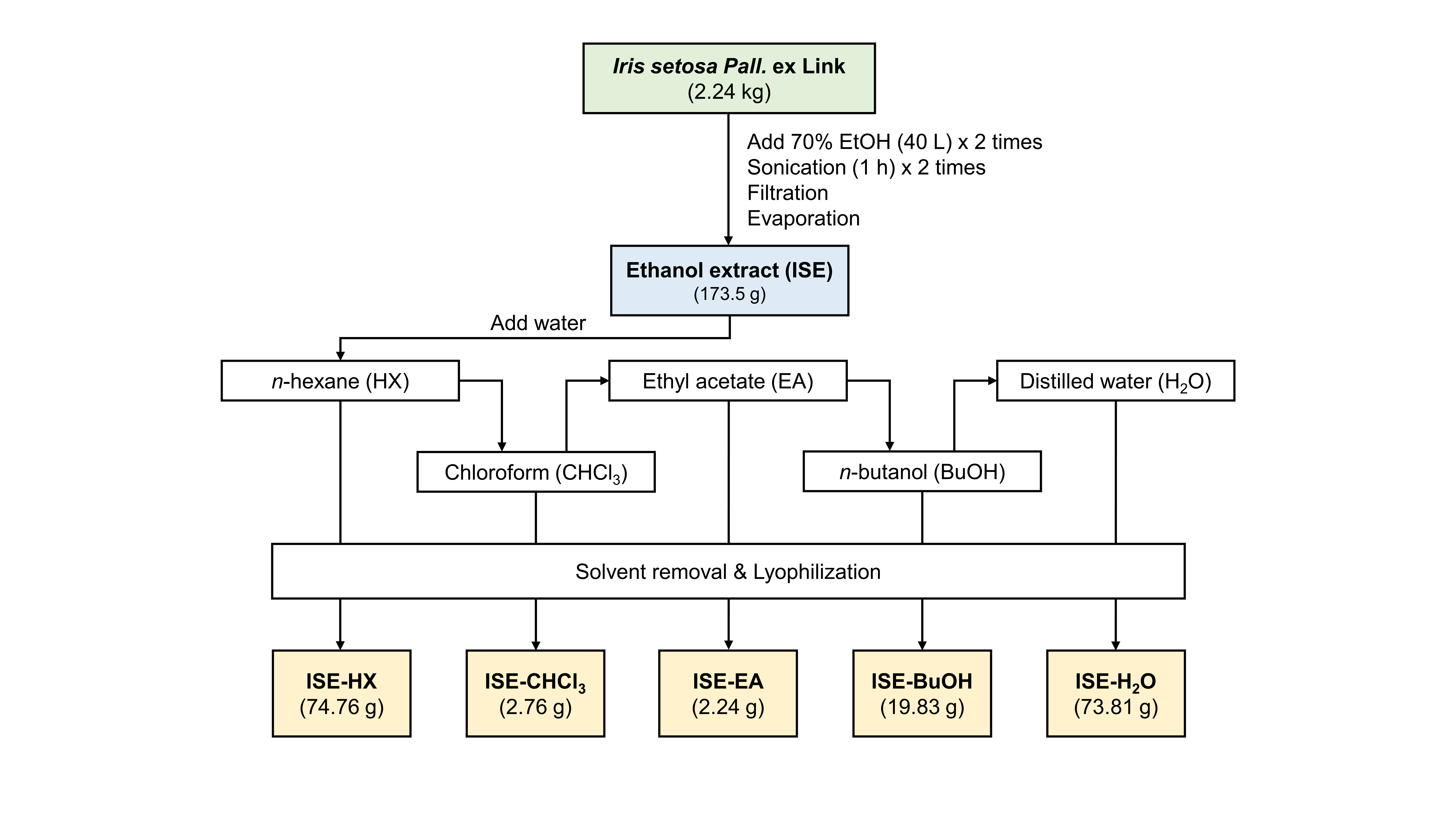

Supplement: Supplementary file 1 [file microorganisms-12-01658-s001.zip › Figure S1.tif]

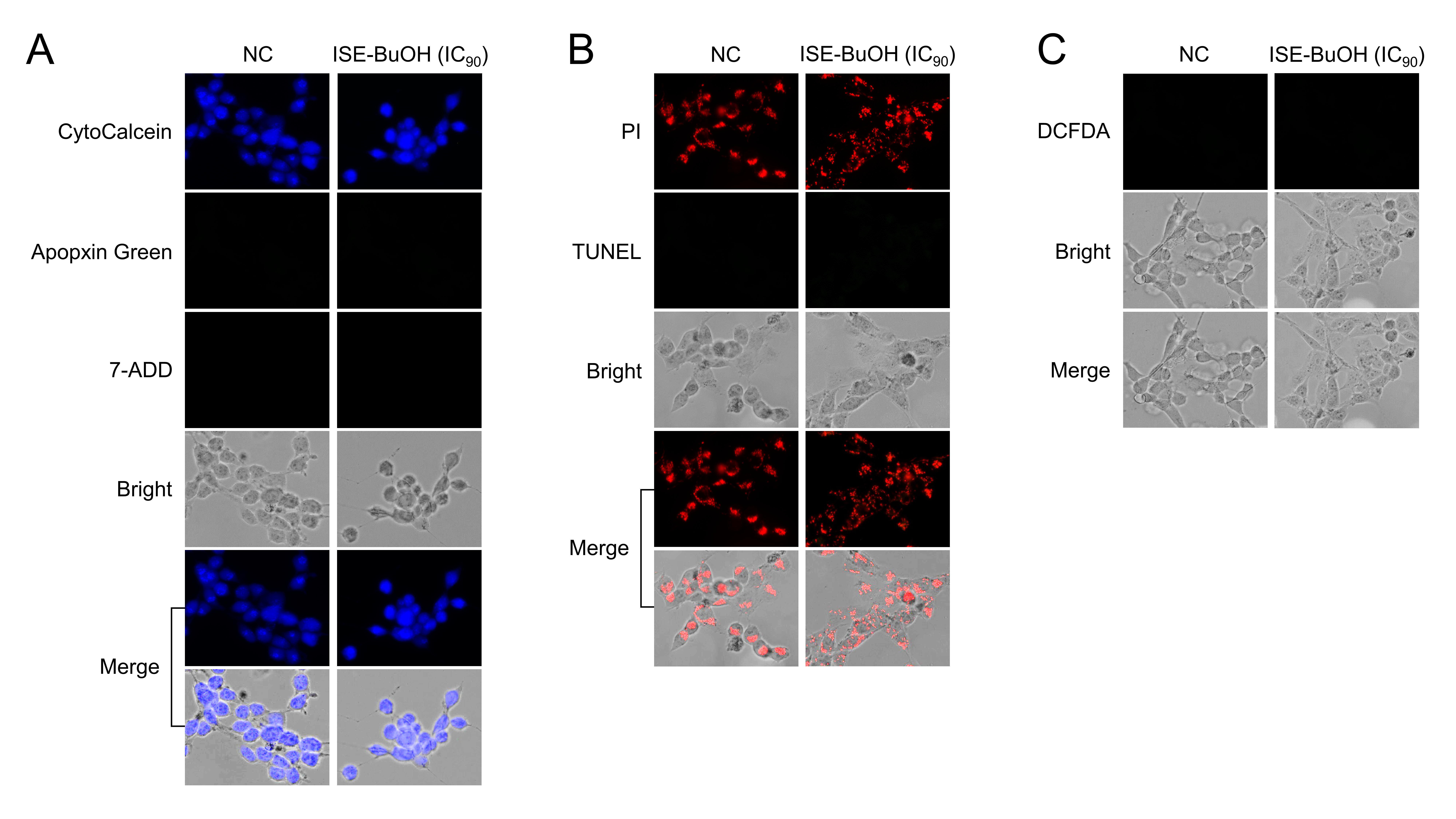

Supplement: Supplementary file 1 [file microorganisms-12-01658-s001.zip › Figure S2.tif]
